# Are *PTTG1* variants associated with tumor characteristics and p53/Ki-67 expression in pituitary neuroendocrine tumors

**DOI:** 10.3389/fendo.2025.1717301

**Published:** 2026-01-07

**Authors:** Ieva Baikstiene, Monika Duseikaite, Alvita Vilkeviciute, Martyna Juskiene, Jurgita Makstiene, Lina Poskiene, Arimantas Tamasauskas, Rasa Verkauskiene, Rasa Liutkeviciene, Birute Zilaitiene

**Affiliations:** 1 Institute of Endocrinology, Department of Endocrinology, Lithuanian University of Health Sciences, Kaunas, Lithuania; 2 Institute of Neuroscience, Lithuanian University of Health Sciences, Kaunas, Lithuania; 3 Department of Pathology, Lithuanian University of Health Sciences, Kaunas, Lithuania

**Keywords:** PTTG1, rs1895320, rs2910200, rs6882742, pituitary neuroendocrine tumor, PitNETs, p53, Ki-67

## Abstract

**Background:**

Pituitary tumor-transforming gene 1 (*PTTG1*) is a proto-oncogene implicated in pituitary neuroendocrine tumors (PitNETs) pathogenesis through regulation of the cell cycle, genomic instability, and angiogenesis. Overexpression of *PTTG1* promotes tumor growth, but the impact of its genetic variants in PitNETs size is insufficiently defined.

**Objective:**

To evaluate the impact of *PTTG1* gene variants (rs1895320, rs2910200, and rs6882742), circulating PTTG1 levels, and immunohistochemical markers (Ki-67 and p53) on PitNETs susceptibility and clinical features.

**Methods:**

case–control study included patients with PitNETs and age- and gender-matched controls. Diagnosis of PitNETs was confirmed by MRI/CT and/or histopathology. Genomic DNA was extracted from peripheral blood, and three *PTTG1* variants (rs1895320, rs2910200, rs3811999) were genotyped using TaqMan^®^ real-time PCR assays. Serum PTTG1 levels were measured by ELISA, while Ki-67 and p53 expression were assessed immunohistochemically with digital image analysis. Statistical analyses included chi-square comparisons of genotype/allele distributions, logistic regression for PitNETs risk (odds ratios, 95% CI), and nonparametric tests for biomarker evaluation.

**Results:**

A total of 340 participants were enrolled, comprising 120 PitNET patients and 220 controls. Median age (53.5 *vs*. 54 years) and gender distribution did not differ between the groups. Among patients, 35% had microadenomas and 65% had macroadenomas. Logistic regression revealed that the *PTTG1* rs3811999 TT genotype was associated with increased odds of microadenoma occurrence (OR = 2.53, 95% CI: 1.17-5.48, p = 0.018). The *PTTG1* rs2910200 TT genotype and T allele were significantly more common in tumors with lower proliferative activity Ki-67 LI < 3% (p = 0.013 and p = 0.004), suggesting a potential association with reduced proliferation. In contrast, the rs3811999 TT genotype and T allele were more frequent in tumors with Ki-67 LI > 3% (p = 0.015 and p = 0.011), indicating a relationship with higher proliferative potential. Macroadenomas exhibited significantly higher p53 H-scores than microadenomas (27.34 *vs*. 16.00, p = 0.012), while no associations were observed with gender, invasiveness, activity, or recurrence.

**Conclusions:**

Results suggest that *PTTG1* rs3811999 may influence tumor size or growth pattern, possibly contributing to early tumorigenesis. We can hypothesize that the variant may alter gene expression or protein function, thereby predisposing to PitNETs development at an earlier stage (microadenomas). Future research should integrate molecular studies with larger genetic datasets to clarify how *PTTG1* variants contribute to PitNETs’ pathophysiology.

## 1 Introduction

Pituitary neuroendocrine tumors (PitNETs) are the most common tumor of the pituitary gland, accounting for 10–15% of all intracranial neoplasms (1). Although this neuroendocrine tumor is considered benign, 35% of these tumors are characterized by invasive growth, a more aggressive disease course with a higher probability of recurrence (2, 3). Treatment of tumor recurrence is associated with a higher risk of complications (e.g., hypopituitarism) and higher costs of treatment. The search for biomarkers to assess the invasiveness and progression of PitNETs is currently underway.

Pituitary tumor-transforming gene 1 (*PTTG1*) plays a critical role in the behavior of PitNETs, particularly in tumorigenesis, cell proliferation, and invasiveness (4–6). As a proto-oncogene, *PTTG1* encodes securin, a protein that controls sister chromatid separation during mitosis. When overexpressed in PitNETs, *PTTG1* accelerates cell cycle progression, promotes genetic instability, and activates the expression of growth factors such as fibroblast growth factor 2 (FGF-2) and vascular endothelial growth factor (VEGF), driving angiogenesis and enhancing tumor invasion (7, 8). These oncogenic effects may be mediated through multiple molecular pathways, including inhibition of sister chromatid separation, suppression of deoxyribonucleic acid (DNA) double- strand break repair, and transcriptional activation or repression (9). Studies have shown that *PTTG1* expression is significantly upregulated in a wide range of tumors, especially those arising from the endocrine system. Nonetheless, elevated PTTG1 levels have also been observed in non- endocrine cancers, including those of the central nervous system (10, 11), lungs (12, 13), and gastrointestinal tract (14–16).

Elevated expressions of PTTG1 and Ki-67 are linked to higher tumor grade, greater proliferative activity, and poorer clinical outcomes in meningiomas and prostate cancer (17, 18). In meningiomas, PTTG1 shows similar results as Ki-67 for identifying higher grade tumors and can be a useful marker to identify patients with a higher risk. In prostate cancer, a higher PTTG1 level is an independent sign of poor outcome and relates to p53 changes and higher Ki-67, which means it may help for risk grouping and for choosing stronger treatment when needed. In head and neck squamous cell carcinoma, PTTG1 works together with p53 and PTTG1-binding factor (PBF), changing p53 signaling, and this can affect how the tumor grows and how long patients survive. High PTTG1 levels in tumors and problems with p53 are linked to worse results, so looking at both markers together may help to predict outcome and choose treatment (19).

Ye et al., through a genome-wide association study, reported four loci: rs2359536, rs10828088, rs10763170, and rs17083838, as common variants linked to increased risk of sporadic PitNETs in the Han Chinese population (20). The association of rs17083838 was further confirmed in a Portuguese cohort (21). While these variants demonstrate inherited genetic susceptibility to PitNETs, they offer no direct guidance regarding tumor behavior, recurrence, or clinical management. Data from a case-control study in a Chinese population showed that the *PTTG1* rs2910200 variant, in the dominant model and with the T allele, may increase the risk of non-functioning pituitary adenoma (NFPA) (22).

This study aims to investigate the effects of *PTTG1* gene variants (rs1895320, rs2910200, and rs6882742), serum PTTG1 levels, and immunohistochemical markers (Ki-67 and p53) on the susceptibility and clinical characteristics of PitNETs.

## 2 Results

A total of 340 individuals participated in this case–control study, including 120 patients diagnosed with PitNET and 220 age- and sex-matched controls. The median age of participants did not differ significantly between groups (53.5 years in the PitNET group *vs*. 54 years in controls; p > 0.05). In the PitNET group, microadenomas accounted for 35% (n = 42) and macroadenomas for 65% (n = 78) of cases. Regarding functional status, 55.8% (n = 67) of tumors were hormonally active, whereas 44.2% (n = 53) were non-functioning. Invasive growth was identified in 47.5% (n = 57) of patients, while 52.5% (n = 63) presented non-invasive adenomas. Tumor recurrence was recorded in 24 cases (20%), with the remaining 96 patients (80%) showing no evidence of recurrence. A detailed summary of demographic and clinical parameters is presented in **Table 1**.

**Table 1:** Demographic characteristics of study subjects.

Characteristics	Group		*P*-value
	PitNET, n (%) (n=120)	Control, n (%) (n=220)	
Age median (IQR)	53.5 (22)	54 (22)	0.787^
Gender, n %			
Females	70 (58.3)	116 (52.7)	0.321^^
Males	50 (41.7)	104 (47.3)	
Tumor size, n (%)			
Micro PitNET	42 (35)	–	–
Macro PitNET	78 (65)		
Hormonal activity, n (%)			
Active PitNET	67 (55.8)	–	–
Non-active PitNET	53 (44.2)		
Invasiveness, n (%)			
Invasive PitNET	57 (47.5)	–	–
Non-invasive PitNET	63 (52.5)		
Recurrence, n (%)			
PitNET without recurrence	96 (80)	–	–
PitNET with recurrence	24 (20)		

^Mann-Whitney U test; ^^Pearson Chi-Square test.

Evaluation of *PTTG1* variants (rs1895320, rs2910200, and rs3811999) revealed no statistically significant differences in genotype or allele distribution between patients with PitNET and control subjects (p > 0.05) (**Table 2**). Furthermore, results from the binary logistic regression analysis indicated no statistically significant association between these variants and the overall risk of PitNET (**Supplementary Table S1**).

**Table 2:** Distributions of *PTTG1* (rs1895320, rs2910200, rs3811999) genotypes and alleles in patients with PitNET and control groups.

Gene	Genotype/Allele	PitNET group n (%) (n=120)	Control group n (%) (n=220)	*P*-value
*PTTG1* (rs1895320)	AA	93 (77.5)	171 (77.7)	0.925
	AG	24 (20)	42 (19.1)	
	GG	3 (2.5)	7 (3.2)	
	In total:	120 (100)	220 (100)	
	Allele:			
	A	210 (87.5)	384 (87.3)	0.932
	G	30 (12.5)	56 (12.7)	
*PTTG1* (rs2910200)	CC	53 (44.2)	109 (49.5)	0.623
	CT	54 (45)	88 (40)	
	TT	13 (10.8)	23 (10.5)	
	In total:	120 (100)	220 (100)	
	Allele:			
	C	160 (66. 7)	306 (69.5)	0.439
	T	80 (33.3)	134 (30.5)	
*PTTG1* (rs3811999)	CC	42 (35)	87 (39.5)	
	CT	57 (47.5)	103 (46.8)	0.548
	TT	21 (17.5)	30 (13.6)	
	In total:	120 (100)	220 (100)	
	Allele:			
	C	141 (58.8)	277 (63)	0.281
	T	99 (41.3)	163 (37)	

The distribution of genotypes and alleles and binary logistic regression analysis for the analyzed SNVs were evaluated in the study groups according to gender; however, no statistically significant differences were observed in either the female subgroup (**Supplementary Table S2** and **Supplementary Table S3**) or the male subgroup (**Supplementary Table S4** and **Supplementary Table S5**).

### 2.1 Associations of *PTTG1* (rs1895320, rs2910200, rs3811999) with pituitary neuroendocrine tumors size

PitNETs were classified into microadenomas and macroadenomas for subgroup analysis. Comparison of *PTTG1* variant (rs1895320, rs2910200, rs3811999) genotype and allele frequencies among microadenoma, macroadenoma, and control groups showed no statistically significant differences across the analyzed groups (p > 0.05) (**Table 3**).

**Table 3:** Distributions of *PTTG1* (rs1895320, rs2910200, rs3811999) genotypes and alleles in PitNET and control groups by PitNET size.

Gene	Genotype/Allele	Control group n (%) (n=220)	Micro PitNET (n=42) n (%)	*p*-value	Macro PitNET (n=78) n (%)	*p*-value
*PTTG1* (rs1895320)	AA	171 (77.7)	33 (78.6)	0.487	60 (76.9)	0.960
	AG	42 (19.1)	9 (21.4)		15 (19.2)	
	GG	7 (3.2)	0 (0)		3 (3.8)	
	In total:	220 (100)	42 (100)		78 (100)	
	Allele:					
	A	384 (87.3)	75 (89.3)	0.608	135 (86.5)	0.814
	G	56 (12.7)	9 (10.7)		21 (13.5)	
*PTTG1* (rs2910200)	CC	109 (49.5)	20 (47.6)	0.423	33 (42.3)	0.476
	CT	88 (40)	20 (47.6)		34 (43.6)	
	TT	23 (10.5)	2 (4.8)		11 (14.1)	
	In total:	220 (100)	42 (100)		78 (100)	
	Allele:					
	C	306 (69.5)	60 (71.4)	0.730	100 (64.1)	0.210
	T	134 (30.5)	24 (28.6)		56 (35.9)	
*PTTG1* (rs3811999)	CC	87 (39.5)	15 (35.7)	0.050	27 (34.6)	0.564
	CT	103 (46.8)	15 (35.7)		42 (53.8)	
	TT	30 (13.6)	12 (28.6)		9 (11.5)	
	In total:	220 (100)	42 (100)		78 (100)	
	Allele:					
	C	277 (63)	45 (53.6)	0.105	96 (61.5)	0.753
	T	163 (37)	39 (46.4)		60 (38.5)	

Binary logistic regression analysis was also performed for microadenomas and macroadenomas in comparison with the control group. The results showed that the *PTTG1* rs3811999 TT genotype, compared with CC + CT, under the most robust genetic model (selected based on the lowest AIC value), was associated with 2.5-fold increased odds of microadenoma occurrence (OR 2.533; 95% CI: 1.170-5.484; p = 0.018) (**Table 4**).

**Table 4:** Binary logistic regression analysis of *PTTG1* (rs1895320, rs2910200, rs3811999) in the PitNET and control groups by PitNET size.

Model	Genotype/Allele	OR (95% CI)	*P*-value	AIC
*PTTG1* rs1895320				
Micro PitNET				
Codominant	AG *vs.* AA GG *vs.* AA	1.110 (0.494-2.498) -	0.800 -	232.106
Dominant	AG+GG *vs.* AA	0.952 (0.427-2.124)	0.904	232.637
Recessive	GG *vs.* AA+AG	–	–	230.170
Overdominant	AG *vs.* AA+GG	1.156 (0.514-2.599)	0.726	235.532
Additive	G	0.837 (0.410-1.709)	0.626	232.405
Macro PitNET				
Codominant	AG *vs.* AA GG *vs.* AA	1.018 (0.527-1.967) 1.221 (0.306-4.875)	0.958 0.777	346.546
Dominant	AG+GG *vs.* AA	1.047 (0.566-1.937)	0.884	344.604
Recessive	GG *vs.* AA+AG	1.217 (0.307-4.828)	0.780	344.549
Overdominant	AG *vs.* AA+GG	1.009 (0.524-1.944)	0.978	344.624
Additive	G	1.058 (0.64-1.747)	0.827	344.577
*PTTG1* rs2910200				
Micro PitNET				
Codominant	CT *vs.* CC TT *vs.* CC	1.239 (0.627-2.446) 0.474 (0.103-2.170)	0.538 0.336	232.727
Dominant	CT+TT *vs.* CC	1.080 (0.558-2.091)	0.819	232.600
Recessive	TT *vs.* CC+CT	0.428 (0.097-1.890)	0.263	231.106
Overdominant	CT *vs.* CC+TT	1.364 (0.703-2.646)	0.359	231.816
Additive	T	0.915 (0.550-1.524)	0.733	232.535
Macro PitNET				
Codominant	CT *vs.* CC TT *vs.* CC	1.276 (0.732-2.224) 1.580 (0.698-3.557)	0.389 0.273	345.155
Dominant	CT+TT *vs.* CC	1.339 (0.795-2.255)	0.272	343.411
Recessive	TT *vs.* CC+CT	1.406 (0.651-3.037)	0.386	343.896
Overdominant	CT *vs.* CC+TT	1.159 (0.687-1.955)	0.580	344.319
Additive	T	1.262 (0.867-1.837)	0.224	343.158
*PTTG1* rs3811999				
Micro PitNET				
Codominant	CT *vs.* CC TT *vs.* CC	0.845 (0.391-1.825) 2.320 (0.977-5.511)	0.668 0.057	229.325
Dominant	CT+TT *vs.* CC	1.177 (0.593-2.340)	0.641	232.432
Recessive	TT *vs.* CC+CT	2.533 (1.170-5.484)	**0.018**	227.510
Overdominant	CT *vs.* CC+TT	0.631 (0.318-1.251)	0.187	230.868
Additive	T	1.448 (0.912-2.298)	0.116	230.191
Macro PitNET				
Codominant	CT *vs.* CC TT *vs.* CC	1.314 (0.749-2.304) 0.967 (0.409-2.287)	0.341 0.938	345.480
Dominant	CT+TT *vs.* CC	1.047 (0.566-1.937)	0.884	344.604
Recessive	TT *vs.* CC+CT	1.217 (0.307-4.828)	0.780	344.549
Overdominant	CT *vs.* CC+TT	1.009 (0.524-1.944)	0.978	344.624
Additive	T	1.065 (0.725-1.563)	0.749	344.523

OR, odds ratio; CI, confidence interval; AIC, Akaike information criteria; p-value: significance level (statistically significant when p < 0.05).

### 2.2 Associations of *PTTG1* (rs1895320, rs2910200, rs3811999) with pituitary neuroendocrine tumor invasiveness

The relationship between adenoma invasiveness and genotype/allele distribution was assessed by comparing non-invasive and invasive PitNET groups with the control group. The analysis of *PTTG1* (rs1895320, rs2910200, rs3811999) showed no statistically significant differences in genotype or allele frequencies across the groups (**Table 5**).

**Table 5:** Distributions of *PTTG1* (rs1895320, rs2910200, rs3811999) genotypes and alleles in PitNET and control groups by PitNET invasiveness.

Gene	Genotype/ Allele	Control group n (%) (n=220)	Non- invasive PitNET (n=63) n (%)	*p*-value	Invasive PitNET (n=57) n (%)	*p*-value
*PTTG1* (rs1895320)	AA	171 (77.7)	47 (74.6)	0.859	46 (80.7)	0.807
	AG	42 (19.1)	14 (22.2)		10 (17.5)	
	GG	7 (3.2)	2 (3.2)		1 (1.8)	
	In total:	220 (100)	63 (100)		57 (100)	
	Allele:					
	A	384 (87.3)	108 (85.7)	0.647	102 (89.5)	0.523
	G	56 (12.7)	18 (14.3)		12 (10.5)	
*PTTG1* (rs2910200)	CC	109 (49.5)	26 (41.3)	0.365	28 (49.1)	0.998
	CT	88 (40)	28 (44.4)		23 (40.4)	
	TT	23 (10.5)	9 (14.3)		6 (10.5)	
	In total:	220 (100)	63 (100)		57 (100)	
	Allele:					
	C	306 (69.5)	80 (63.5)	0.198	79 (69.3)	0.959
	T	134 (30.5)	46 (36.5)		35 (30.7)	
*PTTG1* (rs3811999)	CC	87 (39.5)	25 (39.7)	0.946	16 (28.1)	0.183
	CT	103 (46.8)	31 (49.2)		29 (50.9)	
	TT	30 (13.6)	7 (11.1)		12 (21.1)	
	In total:	220 (100)	63 (100)		57 (100)	
	Allele:					
	C	277 (63)	81 (64.3)	0.784	61 (53.5)	0.065
	T	163 (37)	45 (35.7)		53 (46.5)	

A binary logistic regression analysis between the non-invasive PitNET group and the control group, as well as between the invasive PitNET group and the control group of *PTTG1* (rs1895320, rs2910200, rs3811999), did not show any statistically significant results (**Supplementary Table S6**).

### 2.3 Associations of *PTTG1* (rs1895320, rs2910200, rs3811999) with pituitary neuroendocrine tumor activity

PitNETs was also divided into active and non-active groups. After evaluating the distribution of genotypes and alleles of *PTTG1* (rs1895320, rs2910200, rs3811999) in hormonal non- active/active PitNET and the control groups, the analysis revealed no statistically significant differences between the groups (**Table 6**).

**Table 6:** Distributions of *PTTG1* (rs1895320, rs2910200, rs3811999) genotypes and alleles in patients with PitNET and control groups by PitNETs activity.

Gene	Genotype/Allele	Control group n (%) (n=220)	Non- active PitNET (n=53) n (%)	*p*-value	Active PitNET (n=67) n (%)	*p*-value
*PTTG1* (rs1895320)	AA	171 (77.7)	43 (81.1)	0.417	50 (74.6)	0.820
	AG	42 (19.1)	10 (18.9)		14 (20.9)	
	GG	7 (3.2)	0 (0)		3 (4.5)	
	In total:	220 (100)	53 (100)		67 (100)	
	Allele:					
	A	384 (87.3)	96 (90.6)	0.350	114 (85.1)	0.510
	G	56 (12.7)	10 (9.4)		20 (14.9)	
*PTTG1* (rs2910200)	CC	109 (49.5)	25 (47.2)	0.447	28 (41.8)	0.433
	CT	88 (40)	25 (47.2)		29 (43.3)	
	TT	23 (10.5)	3 (5.7)		10 (14.9)	
	In total:	220 (100)	53 (100)		67 (100)	
	Allele:					
	C	306 (69.5)	75 (70.8)	0.807	85 (63.4)	0.183
	T	134 (30.5)	31 (29.2)		49 (36.6)	
*PTTG1* (rs3811999)	CC	87 (39.5)	14 (26.4)	0.153	28 (41.8)	0.876
	CT	103 (46.8)	28 (52.8)		29 (43.3)	
	TT	30 (13.6)	11 (20.8)		10 (14.9)	
	In total:	220 (100)	53 (100)		67 (100)	
	Allele:					
	C	277 (63)	56 (52.8)	0.055	85 (63.4)	0.919
	T	163 (37)	50 (47.2)		49 (36.6)	

Similarly to previous results, a binary logistic regression analysis between the non-active/active PitNET group and the control group of *PTTG1* (rs1895320, rs2910200, rs3811999) did not show any statistically significant results (**Supplementary Table S7**).

### 2.4 Associations of *PTTG1* (rs1895320, rs2910200, rs3811999) with pituitary neuroendocrine tumor recurrence

All patients with PitNETs were also divided into PitNET without recurrence and PitNET with recurrence groups. After evaluating the distribution of genotypes and alleles of *PTTG1* (rs1895320, rs2910200, rs3811999) in PitNET without recurrence and PitNET with recurrence, and the control groups, the analysis revealed no statistically significant differences between the groups (**Table 7**).

**Table 7:** Distributions of *PTTG1* (rs1895320, rs2910200, rs3811999) genotypes and alleles in patients with PitNET and control groups by PitNET recurrence.

Gene	Genotype/Allele	Control group n (%) (n=220)	PitNET without recurrence (n=96) n (%)	*P*-value	PitNET with recurrence (n=24) n (%)	*P*-value
*PTTG1* (rs1895320)	AA	171 (77.7)	73 (76)	0.938	20 (83.3)	0.632
	AG	42 (19.1)	20 (20.8)		4 (16.7)	
	GG	7 (3.2)	3 (3.1)		0 (0)	
	In total:	220 (100)	96 (100)		24 (100)	
	Allele:					
	A	384 (87.3)	166 (86.5)	0.779	44 (91.7)	0.378
	G	56 (12.7)	26 (13.5)		4 (8.3)	
*PTTG1* (rs2910200)	CC	109 (49.5)	45 (46.9)	0.897	8 (33.3)	0.313
	CT	88 (40)	41 (42.7)		13 (54.2)	
	TT	23 (10.5)	10 (10.4)		3 (12.5)	
	In total:	220 (100)	96 (100)		24 (100)	
	Allele:					
	C	306 (69.5)	131 (68.2)	0.741	29 (60.4)	0.195
	T	134 (30.5)	61 (31.8)		19 (39.6)	
*PTTG1* (rs3811999)	CC	87 (39.5)	36 (37.5)	0.645	6 (25)	0.379
	CT	103 (46.8)	43 (44.8)		14 (58.3)	
	TT	30 (13.6)	17 (17.7)		4 (16.7)	
	In total:	220 (100)	96 (100)		24 (100)	
	Allele:					
	C	277 (63)	115 (59.9)	0.466	26 (54.2)	0.233
	T	163 (37)	77 (40.1)		22 (45.8)	

Binary logistic regression analysis comparing the PitNET groups without and with recurrence to the control group for studied SNVs revealed no statistically significant associations. This finding is consistent with previous analyses, which also showed no significant associations (**Supplementary Table S8**).

#### 2.4.1 Serum PTTG1 levels in patients with PitNET and controls

**Figure 1:**
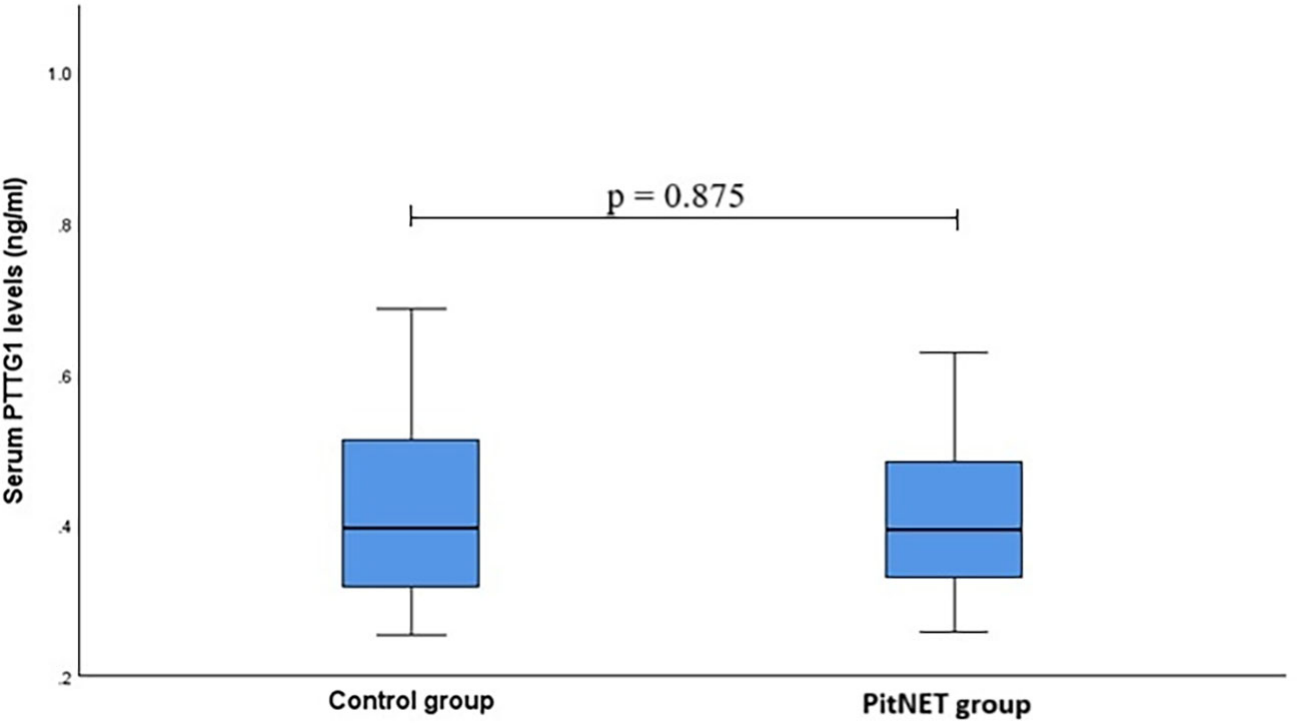
Serum PTTG1 levels.

The study evaluated serum levels of PTTG1 in patients with PitNET compared to a control group. However, no statistically significant differences were found between the serum PTTG1 levels in PitNET patients and the control group. For PTTG1 levels: PitNET patients *vs*. control group: median (IQR):0.396 0.195) ng/mL *vs*. 0.392 (0.171) ng/mL, p = 0.875 (**Figure 1**).

#### 2.4.2 Ki-67 labeling index

In this part of the study, the 69 PitNET tissue samples were analyzed. The Ki-67 LI was evaluated in 40 females (58%) and 29 males (42%). The results revealed no statistically significant differences in the Ki-67 LI between females and males (p = 0.273). Immunohistochemistry for Ki-67 revealed an LI < 3% in 66.7% of patients with PitNET and a Ki-67 LI > 3% in 23% of patients. Further analyses revealed no statistical significance concerning tumor size (p = 0.284), invasiveness (p = 0.125), activity (p = 0.496), or recurrence (p = 0.561). Results are shown in **Table 8**.

**Table 8:** Ki-67 LI, considering the characteristics of PitNET.

Characteristics		Ki-67 LI		*P*-value
		<3%	>3%	
Tumor size	Micro PitNET (n = 24) (%)	18 (75)	6 (25)	0.284
	Macro PitNET (n = 45) (%)	28 (62.2)	17 (37.8)	
Invasiveness	Non-invasive PitNET (n = 36) (%)	27 (75)	9 (25)	0.125
	Invasive PitNET (n = 33) (%)	19 (57.6)	14 (42.4)	
Activeness	Non-active PitNET (n = 34) (%)	24 (70.6)	10 (29.4)	0.496
	Active PitNET (n = 35) (%)	22 (62.9)	13 (37.1)	
Recurrence	PitNET without recurrence (n = 51) (%)	35 (68.6)	16 (31.4)	0.561
	PitNET with recurrence (n= 18) (%)	11 (61.1)	7 (38.9)	

After the analysis of the Ki-67 LI with the genetic variations (*PTTG1* rs1895320, rs2910200, and rs3811999) in two Ki-67 LI groups (<3%, n = 46, and >3%, n = 23), we found that the *PTTG1* rs2910200 TT genotype and T allele were statistically significantly more frequent in the tumors with Ki-67 LI < 3% compared to the Ki-67 LI > 3% (15.2% vs. 4.3%, p = 0.013 and 41.3% vs. 17.4%, p = 0.004, respectively). Also, we found that the *PTTG1* rs3811999 TT genotype and T allele were statistically significantly more frequent in the tumors with Ki-67 LI > 3% compared to the Ki-67 LI < 3% (34.8% vs. 8.7%, p = 0.015 and 60.9% vs. 38%, p = 0.011, respectively) (**Table 9**).

**Table 9:** Ki-67 LI associations with *PTTG1* (rs1895320, rs2910200, rs3811999).

Gene, SNV	Genotype/Allele	Ki-67 LI		*p*-Value
		<3%	>3%	
*PTTG1* rs1895320	AA	39 (84.8)	17 (73.9)	0.273
	AG	7 (15.2)	5 (21.7)	
	GG	0 (0)	1 (4.3)	
	In total:	46 (100)	23 (100)	
	Allele			
	A	85 (92.4)	39 (84.8)	0.162
	G	7 (7.6)	7 (15.2)	
*PTTG1* rs2910200	CC	15 (32.6)	16 (69.6)	**0.013**
	CT	24 (52.2)	6 (26.1)	
	TT	7 (15.2)	1 (4.3)	
	In total:	46 (100)	23 (100)	
	Allele			
	A	54 (58.7)	38 (82.6)	**0.004**
	T	38 (41.3)	8 (17.4)	
*PTTG1* rs3811999	CC	15 (32.6)	3 (13)	**0.015**
	CT	27 (8.7)	12 (52.2)	
	TT	4 (8.7)	8 (34.8)	
	In total:	46 (100)	23 (100)	
	Allele			
	C	57 (62)	18 (39.1)	**0.011**
	T	35 (38)	28 (60.9)	

Bold values indicate statistically significant results.

#### 2.4.3 p53 analysis

A total of 57 PitNET tissue samples were examined for p53 expression. The cohort included 30 female (52.6%) and 27 male (47.4%) patients. Comparison of p53 H-scores between genders revealed no statistically significant difference (p = 0.342). Immunohistochemical analysis revealed that macroadenomas had statistically significantly higher p53 H-scores than microadenomas (median (IQR): 27.34 (24.83) *vs*. 16.00 (16.83); p = 0.012). No additional associations were identified between p53 expression and tumor invasiveness, hormonal activity, or recurrence status (**Table 10**).

**Table 10:** Associations of clinical features of PitNET with p53 H-score.

PitNET subgroups	p53 H-score median (IQR)	*P*-value
Micro PitNET	16 (16.83)	**0.012**
Macro PitNET	27.34 (24.83)	
Non-invasive PitNET	22.66 (18.75)	0.632
Invasive PitNET	26.33 (24.14)	
Non-active PitNET	20.34 (16.84)	0.092
Active PitNET	28.16 (38.86)	
PitNET without recurrence	26.33 (25.16)	0.183
PitNET with recurrence	18.83 (16.99)	

*Mann-Whitney U test was used; PitNET, pituitary neuroendocrine tumor.

**Figure 2:**
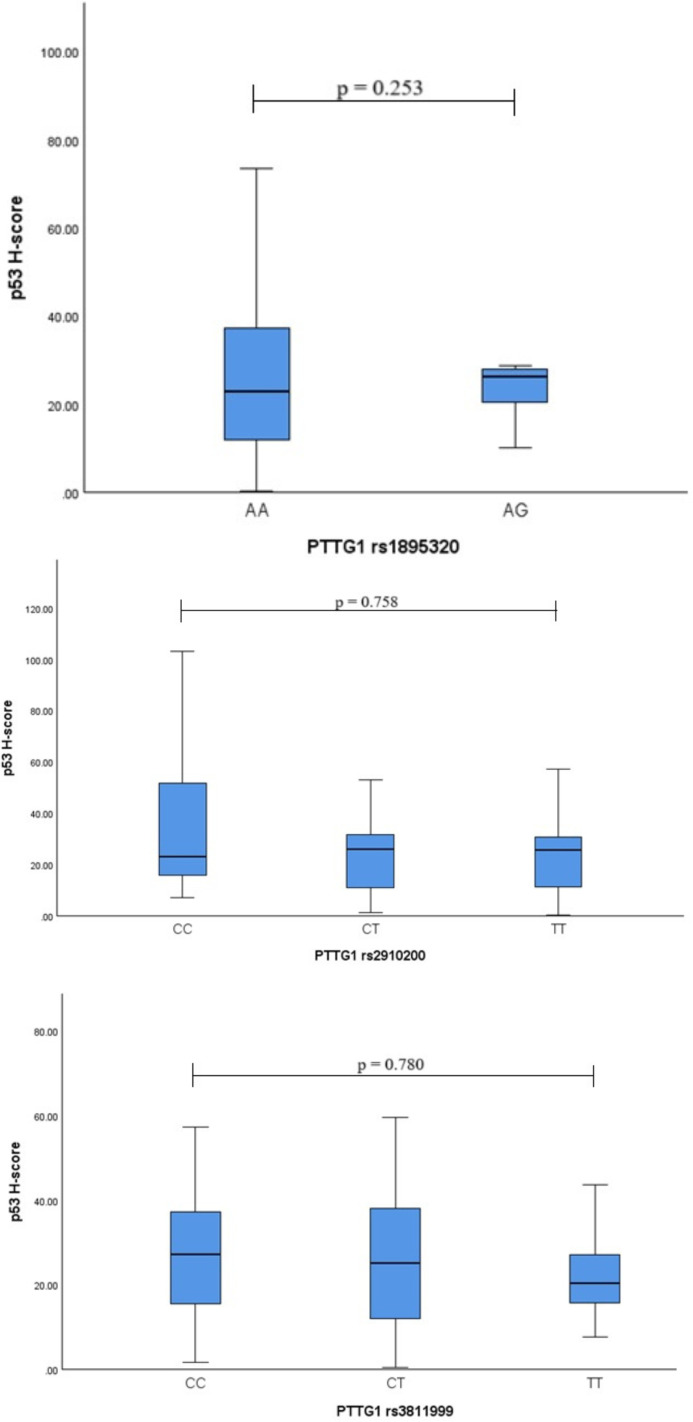
*PTTG1* variants genotype associations with p53 H-score.

To assess the association of the *PTTG1* (rs1895320, rs2910200, rs3811999) variants with p53, the p53 H-score was calculated in different genotype groups, but no statistically significant differences were found (**Figure 2**) (Kruskal-Wallis test was used).

#### 2.4.4 Correlation between Ki-67 and p53 expression

**Figure 3:**
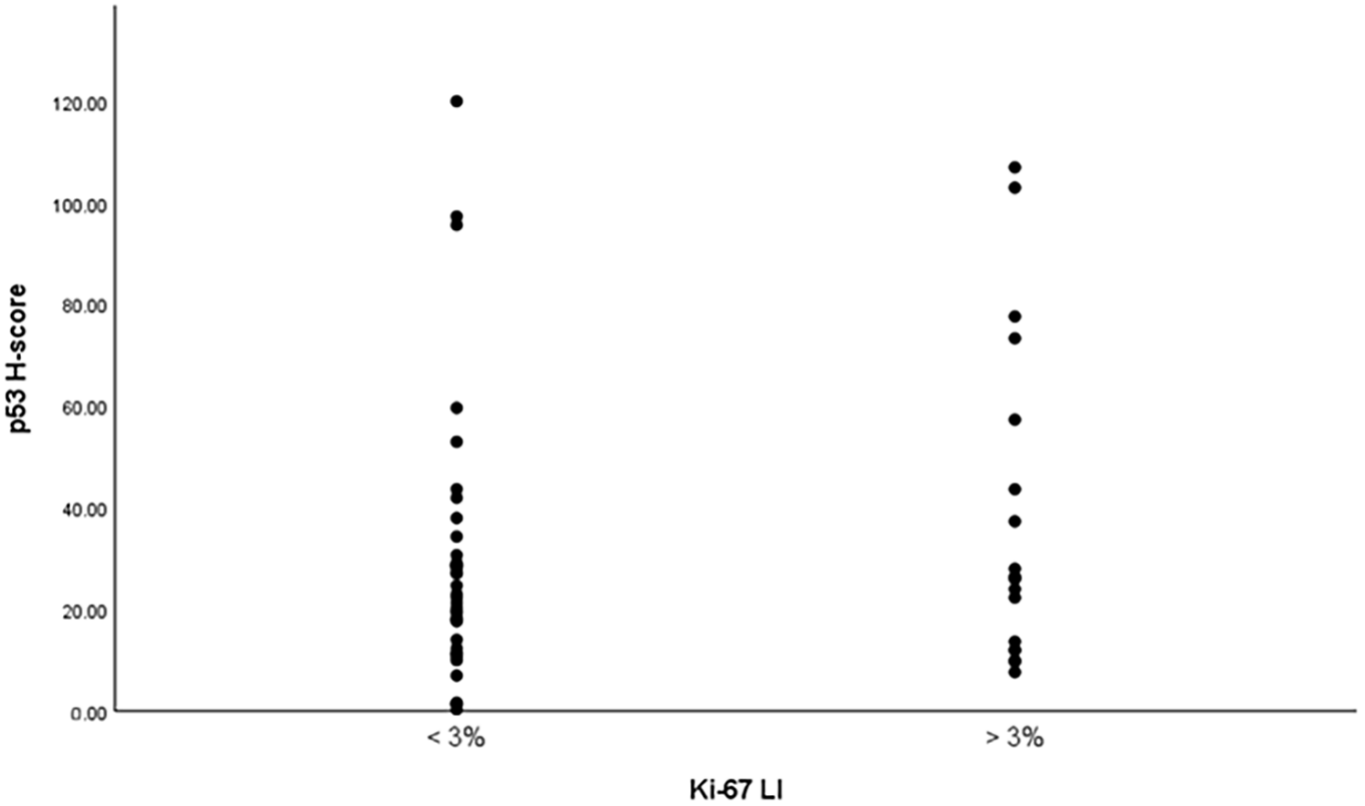
Correlation between Ki-67 LI and p53 H-score.

A nonparametric Spearman’s rank-order correlation analysis was conducted to examine the association between Ki-67 LI and p53 expression across 57 PitNET tissue samples. The analysis demonstrated a weak positive correlation (ρ = 0.105), which did not reach statistical significance (ρ = 0.436). The 95% confidence interval for Spearman’s rho ranged from -0.153 to 0.348. These findings indicate that no significant association between p53 and Ki-67 expression was observed in this cohort (**Figure 3**).

## 3 Discussion

*PTTG1* is an oncogene involved in cell cycle regulation, DNA repair, and chromosomal stability. It is minimally expressed in normal pituitary tissue but is markedly upregulated in most PitNETs, including nonfunctioning and hormone-secreting subtypes (9). Overexpression has been linked to tumor growth, invasiveness, and recurrence in PitNET (5, 23). Our study demonstrates that the rs3811999 TT genotype was associated with increased microadenoma risk (OR 2.533; 95% CI: 1.170-5.484; p = 0.018), suggesting a possible role for *PTTG1* variation. We can hypothesize that the rs3811999 variant may alter gene expression or protein function, thereby predisposing to PitNET development at an earlier stage (microadenomas). The results of this study indicate that genetic variation in *PTTG1* may influence tumor size or growth pattern, possibly contributing to early tumorigenesis. Further research integrating molecular data and larger genetic cohorts is needed to clarify the contribution of *PTTG1* variants to PitNET pathophysiology.

Our finding that the rs3811999 TT genotype was associated with a higher risk of microadenoma contrasts with a previous case–control study in a Han Chinese population, which genotyped five *PTTG1* haplotype-tagging SNVs (including rs3811999) in 280 PitNET patients and 280 matched controls (24). This study reported no significant differences in allele or genotype frequencies of rs3811999 between cases and controls, suggesting that this variant does not increase overall susceptibility to PitNET. The discrepancy between the two studies may reflect differences in study design, sample size, ethnic background, or subgroup stratification (e.g., microadenomas versus macroadenomas), and highlights the need for replication in larger and more diverse cohorts.

In our study, *PTTG1* rs1895320 and rs2910200 did not show statistically significant associations with PitNET size, recurrence, or invasiveness. According to a case-control study in a Chinese Han population, there was no significant association between rs1895320 and susceptibility to NFPA under allelic, dominant, recessive, or additive genetic models (22). This contrasts with other *PTTG1* variants, such as the dominant model of rs2910200, which did show a significant association with NFPA risk in the same study population. For example, in prostate cancer, pathogenic germline variants in DNA repair genes (*ATM*, *CHEK2*, *PALB2*, *BRCA2*) vary by ancestry; some are enriched or even unique to specific populations, leading to differences in prevalence and risk across groups (25, 26).

The results of different studies on *PTTG1* variants and the risk of PitNET are conflicting. This may be explained by population differences (variation in allele frequencies and haplotype structure between ethnic groups), phenotypic heterogeneity (functioning PitNET vs. NFPA), limited sample sizes, and methodological differences. Therefore, larger studies based on diverse populations and homogeneous cohorts are needed to assess the significance of *PTTG1* genetic variants more reliably.

Pituitary macroadenomas had a statistically significantly higher p53 histological score compared to microadenomas, as demonstrated by immunohistochemical analysis in recent studies. Specifically, a recent case-control study found that macroadenomas exhibited a higher median p53 H-score (median (IQR): 30.33 (28.68)) than microadenomas (median (IQR): 18.34 (17.65)), with the difference reaching statistical significance (p = 0.005) (27). This finding supports the association between increased tumor size and elevated p53 expression.

Additional literature supports that p53 expression is more frequently observed in invasive or larger PitNETs and is associated with higher proliferative activity as measured by Ki-67 LI (28, 29). However, the clinical utility of p53 as a prognostic marker remains controversial, as some studies report poor reproducibility and limited predictive value for tumor aggressiveness or recurrence (30–32).

Increased PTTG1 expression is associated with higher tumor proliferation and aggressiveness across multiple tumor types. Several studies have demonstrated a strong correlation between PTTG1 expression and Ki-67, a well-established marker of cellular proliferation. For example, in meningiomas, PTTG1 immunoreactivity closely parallels Ki-67 and is associated with higher tumor grade and proliferation rate (17). Similarly, in PitNETs, PTTG1 and Ki-67 expression are strongly correlated, and higher levels of both markers are linked to increased recurrence risk and more aggressive tumor behavior (33). To date, no studies have directly demonstrated an association between the *PTTG1* rs1895320 G allele and clinical outcomes such as recurrence, progression-free survival, or overall survival in tumors with elevated Ki-67 expression.

Higher Ki-67 expression is strongly associated with more aggressive tumor behavior, increased recurrence risk, and greater invasiveness across multiple tumor types. Ki-67 is a nuclear protein expressed during active phases of the cell cycle and is a validated marker of cellular proliferation. Elevated Ki-67 LI independently predicts poor overall and progression-free survival in gliomas, breast cancer, and meningiomas, and is linked to shorter recurrence-free intervals and earlier time to recurrence (34–36).

Our analysis of proliferative activity, as measured by Ki-67 LI, showed a significant association between *PTTG1* variants and Ki-67 LI. Our results indicate that *PTTG1* rs2910200 and rs3811999 show opposite associations with proliferative activity in PitNETs. The rs2910200 TT genotype and T allele were significantly more common in tumors with low proliferative activity (Ki-67 LI < 3%), suggesting that this variant may be linked to a less proliferative tumor profile. In contrast, the rs3811999 TT genotype and T allele were significantly more frequent in tumors with higher proliferative activity (Ki-67 LI > 3%), indicating a potential association with greater tumor proliferation. This finding is biologically plausible, as *PTTG1* is an oncogene implicated in cell cycle regulation and genomic instability, and its genetic variants may influence tumor growth dynamics. Although Ki-67 is not universally accepted as a prognostic marker in PitNETs, several studies have associated higher Ki-67 expression with more aggressive behavior, recurrence, and invasiveness, indicating that both these variants could serve as potential molecular markers of proliferative activity. Overall, these findings suggest that different *PTTG1* variants may have distinct biological effects on PitNET growth dynamics, with rs2910200 potentially associated with lower proliferation and rs3811999 with higher proliferation. Nevertheless, given the relatively small subgroup sizes and borderline p-value, this result should be interpreted cautiously and validated in larger cohorts with standardized Ki-67 assessment.

In summary, our findings suggest that *PTTG1* variants, particularly rs3811999 TT, which was associated with higher proliferative activity, and rs2910200 TT, which was linked to lower proliferative activity, may differentially influence PitNET susceptibility and proliferative potential. Additionally, the rs3811999 TT genotype may contribute to increased PitNET susceptibility at earlier stages, while p53 expression appears to be linked to tumor size. Although these results provide new insights into the genetic and molecular mechanisms underlying PitNETs’ pathophysiology, they should be interpreted cautiously, given the limited sample size and the heterogeneity across studies. Larger, multicenter investigations integrating genetic, molecular, and clinical data are warranted to validate these associations and clarify their potential prognostic and therapeutic value.

### 3.1 Comparison with publicly available databases

For the *PTTG1* variants examined in our study (rs1895320, rs2910200, and rs3811999), the allele frequencies observed in our control population closely align with those reported for European populations in dbSNP (37). Specifically, the G-allele frequency of rs1895320 shows substantial variation across global populations, ranging from 1.3% to 22.9%, with most large international cohorts reporting values between 11-16%. Our observed frequency of 12.8% aligns well with Northern European datasets such as Northern Sweden, TwinsUK, and ALSPAC. Moreover, the T-allele frequency of rs2910200 ranges from approximately 8-18% in East Asian populations, 20-27% in mixed or global cohorts, and up to 30-35% in Northern Europeans. Our observed T-allele frequency of 30.5% in controls is consistent with these Northern European datasets, including GoNL and Northern Sweden. Similarly, the T-allele frequency of rs3811999 varies markedly across global populations, from ~11-15% in East Asians to ~40-48% in Northern Europeans, with most European and American cohorts reporting values between 25-42%. Our observed frequency of 37% in controls falls within this higher European range and closely matches the ALFA and GENOME_DK datasets. Together, these findings support the validity and representativeness of our genotyping results.

### 3.2 Limitations and future directions

While comparison with publicly available databases strengthens our conclusions, we acknowledge that the borderline significance observed for the Ki-67 associations (p = 0.013, p = 0.004, p = 0.015, and p = 0.011) warrants cautious interpretation. *Post-hoc* power analysis indicates that, given our current sample sizes, particularly within specific subgroups, the study is underpowered, suggesting that this finding should be considered preliminary rather than definitive. This is a common limitation in genetic association studies involving subgroup analyses. Validation in larger, independent cohorts is therefore essential to confirm our observations. Future research should also investigate the functional impact of these *PTTG1* variants on protein expression and cellular phenotypes in PitNET to better elucidate potential causal links between genetic variation and tumor behavior. Despite these limitations, our study, particularly when contextualized with publicly available genomic data, provides important insights into the potential role of *PTTG1* genetic variants in PitNET development and progression.

## 4 Materials and methods

### 4.1 Study design

The Kaunas Regional Biomedical Research Ethics Committee approved the case-control study (Permission No. BE-2-47, issued on 14 December 2016). The research was carried out at the Laboratory of Ophthalmology and the Department of Neurosurgery, Hospital of Lithuanian University of Health Sciences. All participants received a detailed explanation of the study design and objectives, and written informed consent was obtained from everyone in accordance with ethical research standards.

### 4.2 Study population

The study included 120 patients diagnosed with PitNET and a control group of 220 individuals. Controls were selected to match the age and gender distribution of the PitNET group. As a result, the median ages of PitNET patients and control subjects did not differ significantly (p < 0.05). PitNET cases were further categorized into hormonally active and inactive adenomas; however, more detailed stratification into specific functional subtypes (e.g., GH-, ACTH-, or TSH-secreting adenomas) was not performed. In Lithuania, aside from prolactinomas, other functional PitNET subtypes are extremely rare, resulting in subgroup sizes too small for reliable statistical analysis. Using the global PitNET prevalence (20%) and the minor allele frequencies of rs1895320 (G = 14.5%), rs2910200 (T = 27%) and rs3811999 (T = 36%) from the dbSNP database (38), we calculated that our sample sizes (120 PitNET cases and 220 controls) provide less than 80% statistical power, suggesting that future studies should include larger cohorts to achieve sufficient power.

Treatment-related factors (e.g., surgery, radiotherapy, or pharmacotherapy) were not analyzed in this study and were not included as covariates in the statistical models.

Inclusion criteria for patients with **PitNET**:

1. Diagnosis of PitNET confirmed by MRI/CT and/or histopathological examination.
2. Overall good general health.
3. Participation was based on signed informed consent.
4. Participants aged ≥ 18 years.
5. Absence of other brain or localized tumors.

Exclusion criteria for patients with PitNET:

1. No confirmed PitNET diagnosis on imaging (MRI/CT) and/or histopathology.
2. Age <18 years, or significant health conditions likely to affect participation or study outcomes.
3. General health was considered poor by clinical assessment.
4. Presence of other brain tumors, extracranial tumors, intracranial infections, demyelinating lesions, or cerebrovascular disease.
5. No informed consent provided.

Inclusion criteria for the control group:

1. Good general health, without history of PitNET or major medical conditions affecting study outcomes.
2. Age ≥ 18 years to ensure comparability with the patient cohort.
3. No history of brain tumors, extracranial tumors, intracranial infections, demyelinating disease, cerebrovascular disorders, or other systemic illnesses.
4. No prior diagnosis or clinical/imaging evidence of pituitary disorders.
5. Signed written informed consent.

Exclusion criteria for the control group:

1. Presence of significant health conditions, including pituitary disorders, brain tumors, or major systemic diseases.
2. Age <18 years.
3. History of pituitary or brain disorders.
4. Failure to provide informed consent.

### 4.3 DNA extraction and genotyping

Genotyping for *PTTG1* variants (rs1895320, rs2910200, and rs3811999) was carried out at the Laboratory of Ophthalmology, Neuroscience Institute, Lithuanian University of Health Sciences (LUHS). DNA extraction was performed as previously described in our earlier publication (27). Single- nucleotide variants of *PTTG1* (rs1895320, rs2910200, and rs3811999) were carried out using the real-time polymerase chain reaction (RT-PCR) method. TaqMan^®^ Genotyping Assays (Thermo Fisher Scientific, Pleasanton, CA, USA) were used to identify SNVs following the manufacturer’s instructions, using the StepOne Plus system (Applied Biosystems, Waltham, MA, USA) (**Table 11**). To verify accuracy, 5% of the samples were reanalyzed for two SNVs, demonstrating consistent results between the initial and repeat genotyping.

**Table 11:** The specific TaqMan^®^ SNV genotyping assays used for each SNV.

SNV ID	Assay ID	Context sequence [VIC/FAM]
rs1895320	C_11314299_10	TTACTGAATCTCTGCTATTAGGCCT**[A/G]**TCA TAGCCATGCCACTACCAAAAGT
rs2910200	C_26693337_20	GGCATCATCCTACGTAGCTTTCTTC**[C/T]**CTC TGAGTAGAAGGGAAATAGAGGT
rs3811999	C__377089_10	GTAAAAGTAGCTACCATTCCTGCCT**[C/T]**AAT AAAATAGCCCAACATAATAGAA

### 4.4 Serum levels measurement

Peripheral venous blood samples were collected and allowed to clot at room temperature for 30 minutes. Following centrifugation, the serum fraction was carefully separated from cellular components, transferred into 1.5 mL storage tubes, and kept at –80°C until further analysis. Serum PTTG1 concentrations were measured in duplicate for both control and PitNET groups using an enzyme-linked immunosorbent assay (ELISA) with the Human Securin (PTTG1) ELISA Kit (Cat. No. abx259838; detection range: 0.156–10 ng/mL; sensitivity < 0.06 ng/mL). Measurements were performed in accordance with the manufacturer’s protocol using a Multiskan FC Microplate Photometer (Thermo Scientific, Waltham, MA, USA) set to a 450 nm wavelength.

### 4.5 Evaluation of Ki-67 and p53

Assessment of Ki-67 LI and p53 expression was conducted by an experienced pathologist at the Department of Pathological Anatomy, LUHS. Immunohistochemical staining for both biomarkers was performed using the Ventana BenchMark ULTRA PLUS automated staining system (Roche Diagnostics, Basel, Switzerland) in accordance with the manufacturer’s protocol.

Immunohistochemical staining was detected using monoclonal antibodies: Ki-67 (clone SP6, Vitro S.A., Sevilla, Spain) and p53 (clone DO-7, Roche Diagnostics, Basel, Switzerland). After performing Ki-67 and p53 immunohistochemical reactions, the images were digitized with a Pannoramic 250 FLASH III scanner (3DHISTECH Ltd., Budapest, Hungary). The evaluation of the digitized images for Ki-67 and p53 was conducted using the 3DHISTECH SlideViewer 2.9.0. software (3DHISTECH Ltd., Budapest, Hungary), based on the 5^th^ WHO Classification of Endocrine and Neuroendocrine Tumors (39).

The procedures for assessing Ki-67 and p53 immunostaining, including hotspot selection, scoring criteria, and H-score calculation, were conducted as described in our previous publication (27).

### 4.6 Statistical analysis

All statistical analyses were conducted using SPSS for Windows, version 30.0 (IBM Corp., Chicago, IL, USA). Categorical variables were presented as absolute numbers and percentages, while continuous variables were summarized as medians with interquartile ranges (IQRs). Genotype and allele distributions between PitNET patients and controls were also evaluated using the chi-square test. Binary logistic regression analysis was applied to assess the association between *PTTG1* genotypes and PitNET risk, with results expressed as odds ratios (ORs) and corresponding 95% confidence intervals (CIs). The most robust genetic model was determined using the Akaike Information Criterion (AIC), with the model showing the lowest AIC considered optimal. For the analysis of immunohistochemical markers, nonparametric tests were used: the Mann–Whitney U test was employed to compare p53 H-scores between PitNETs subgroups, and the Spearman’s rank- order correlation (ρ) was calculated to assess the relationship between Ki-67 LI and the p53 H- score. No multiple-comparison correction was applied, as analyses were limited to predefined SNVs and markers based on specific hypotheses. A p-value < 0.05 was considered statistically significant.

### 4.7 Database comparison analysis

To strengthen our conclusions and address potential limitations of our sample size, we compared our genetic variant results with publicly available genomic databases. Allele frequencies of *PTTG1* variants (rs1895320, rs2910200, rs3811999) from our control population were compared with those reported indbSNP (37) database, with particular focus on European populations.

## 5 Conclusions

Our findings suggest that *PTTG1* variants, particularly the rs3811999 TT genotype and rs1895320 G genotype, may contribute to PitNET susceptibility and proliferative activity, while higher p53 expression is associated with macroadenomas. These results indicate a possible role of *PTTG1* genetic variation in early tumorigenesis and tumor growth dynamics. However, due to limited sample size and contradictions with previous studies, larger and more diverse cohorts are needed to validate these associations and clarify their prognostic value.

### 5.1 Study relation to previous work

This study is a separate and independent investigation from our previous publication, “Insights into *FGFR4* (rs351855 and rs7708357) Gene Variants, Ki-67 and p53 in Pituitary Adenoma Pathophysiology” (27). While the earlier research focused on fibroblast growth factor receptor 4 (*FGFR4*) gene variants, serum levels, and associations with immunohistochemical markers (Ki- 67 and p53), the current study explores a different molecular target — the pituitary tumor- transforming gene 1 (*PTTG1*) and its variants (rs1895320, rs2910200, and rs3811999). Both genes are implicated in PitNET pathophysiology but act through distinct biological pathways: *FGFR4* regulates growth factor signaling, while *PTTG1* functions as a proto-oncogene influencing chromosomal stability, cell cycle progression, and tumor proliferation.

Importantly, although both studies investigated patients with PitNET and included Ki-67 and p53 immunohistochemistry for comparative purposes, the genetic analysis, molecular targets, and hypotheses differ completely. Furthermore, the study populations are not identical: the current study enrolled a separate cohort of 120 patients and 220 controls, distinct from the 100 patients and 200 controls analyzed in the *FGFR4* study. Thus, the present work constitutes an independent dataset and a progression of our molecular research, aiming to expand understanding of PitNET biology by investigating new candidate genes (*PTTG1*) within the same disease context.

## Data Availability

The datasets presented in this study can be found in online repositories. The names of the repository/repositories and accession number(s) can be found in the article/**Supplementary Material**.
