# Supplementary material for: Are PTTG1 variants associated with tumor characteristics and p53/Ki-67 expression in pituitary neuroendocrine tumors
Source: Front Endocrinol (Lausanne). 2026 Jan 7;16:1717301. doi: 10.3389/fendo.2025.1717301 (PMC12819198; doi:10.3389/fendo.2025.1717301)
Supplement: Supplementary file 1 [file Table1.docx]

SUPPLEMENTARY MATERIAL

**Table S1. Binary logistic regression analysis of** ***PTTG1* (rs1895320, rs2910200, rs3811999) in patients with PitNET and control groups**

| **Model** | **Genotype/Allele** | **OR (95% CI)** | ***p*-value** | **AIC** |
| --- | --- | --- | --- | --- |
| ***PTTG1* (**rs1895320**)** | | | | |
| Codominant | AG *vs.* AA  GG *vs.* AA | 1.051 (0.599-1.842)  0.788 (0.199-3.119) | 0.863  0.734 | 445.330 |
| Dominant | AG+GG *vs.* AA | 1.013 (0.594-1.727) | 0.962 | 443.487 |
| Recessive | GG *vs.* AA+AG | 0.780 (0.198-3.074) | 0.723 | 443.359 |
| Overdominant | AG *vs.* AA+GG | 1.060 (0.605-1.854) | 0.840 | 443.448 |
| Additive | G | 0.982 (0.627-1.537) | 0.936 | 443.482 |
| ***PTTG1* (**rs2910200**)** | | | | |
| Codominant | CT *vs.* CC  TT *vs.* CC | 1.262 (0.787-2.023)  1.162 (0.546-2.474) | 0.334  0.696 | 444.542 |
| Dominant | CT+TT *vs.* CC | 1.241 (0.794-1.941) | 0.343 | 442.587 |
| Recessive | TT *vs.* CC+CT | 1.041 (0.507-2.137) | 0.914 | 443.477 |
| Overdominant | CT *vs.* CC+TT | 1.227 (0.783-1.924) | 0.372 | 442.693 |
| Additive | T | 1.137 (0.816-1.584) | 0.447 | 442.913 |
| ***PTTG1*** (rs3811999) | | | | |
| Codominant | CT *vs.* CC  TT *vs.* CC | 11.46 (0.702-1.872)  1.450 (0.743-2.829) | 0.585  0.276 | 444.297 |
| Dominant | CT+TT *vs.* CC | 1.125 (0.765-1.929) | 0.409 | 442.804 |
| Recessive | TT *vs.* CC+CT | 1.343 (0.731-2.468) | 0.341 | 442.596 |
| Overdominant | CT *vs.* CC+TT | 1.028 (0.658-1.605) | 0.904 | 443.474 |
| Additive | T | 1.192 (0.864-1.644) | 0.284 | 442.340 |

OR: odds ratio; CI: confidence interval; AIC: Akaike information criteria; p-value: significance level (statistically significant when p < 0.05).

**Table S2. Distributions of *PTTG1* (rs1895320, rs2910200, rs3811999) genotypes and alleles in female patients with PitNET and the control group**

| **Gene** | **Genotype/Allele** | **PitNET group females (n=70)**  **n (%)** | **Control group**  **females (n=116)**  **n (%)** | ***p*-value** |
| --- | --- | --- | --- | --- |
| *PTTG1*  (rs1895320) | AA | 53 (75.7) | 90 (77.6) | 0.950 |
|  | AG | 14 (20) | 21 (18.1) |  |
|  | GG | 3 (4.3) | 5 (4.3) |  |
|  | In total: | 70 (100) | 116 (100) |  |
|  | Allele:  A  G | 120 (85.7)  20 (14.3) | 201 (86.6)  31 (13.4) | 0.801 |
| *PTTG1*  (rs2910200) | CC | 30 (42.9) | 58 (50) | 0.288 |
|  | CT | 35 (50) | 45 (38.8) |  |
|  | TT | 5 (7.1) | 13 (11.2) |  |
|  | In total: | 70 (100) | 116 (100) |  |
|  | Allele:  C  T | 95 (67.9)  45 (32.1) | 161 (69.4)  71 (30.6) | 0.756 |
| *PTTG1*  (rs3811999) | CC | 22 (31.4) | 49 (42.2) | 0.255 |
|  | CT | 38 (54.3) | 49 (42.2) |  |
|  | TT | 10 (14.3) | 18 (15.5) |  |
|  | In total: | 70 (100) | 116 (100) |  |
|  | Allele:  C  T | 82 (58.6)  58 (41.4) | 147 (63.4)  85 (36.6) | 0.357 |

**Table S3. Binary logistic regression analysis of *PTTG1* (rs1895320, rs2910200, rs3811999) in female patients with PitNET and the control group**

| **Model** | **Genotype/Allele** | **OR (95% CI)** | ***p*-value** | **AIC** |
| --- | --- | --- | --- | --- |
| ***PTTG1* (**rs1895320**)** | | | | |
| Codominant | AG *vs.* AA  GG *vs.* AA | 1.132 (0.531-2.413)  1.019 (0.234-4.436) | 0.748  0.980 | 250.253 |
| Dominant | AG+GG *vs.* AA | 1.110 (0.552-2.234) | 0.769 | 248.270 |
| Recessive | GG *vs.* AA+AG | 0.994 (0.230-4.294) | 0.994 | 248.355 |
| Overdominant | AG *vs.* AA+GG | 1.131 (0.533-2.400) | 0.749 | 248.253 |
| Additive | G | 1.066 (0.614-1.852) | 0.819 | 248.303 |
| ***PTTG1* (**rs2910200**)** | | | | |
| Codominant | CT *vs.* CC  TT *vs.* CC | 1.504 (0.806-2.807)  0.744 (0.242-2.283) | 0.200  0.605 | 247.849 |
| Dominant | CT+TT *vs.* CC | 1.333 (0.734-2.422) | 0.345 | 247.460 |
| Recessive | TT *vs.* CC+CT | 0.609 (0.208-1.790) | 0.368 | 247.498 |
| Overdominant | CT *vs.* CC+TT | 1.578 (0.867-2.872) | 0.136 | 246.124 |
| Additive | T | 1.074 (0.684-1.688) | 0.756 | 248.529 |
| ***PTTG1*** (rs3811999) | | | | |
| Codominant | CT *vs.* CC  TT *vs.* CC | 1.727 (0.895-3.334)  1.237 (0.492-3.112) | 0.103  0.651 | 247.608 |
| Dominant | CT+TT *vs.* CC | 1.596 (0.854-2.980) | 0.143 | 246.167 |
| Recessive | TT *vs.* CC+CT | 0.907 (0.939-2.096) | 0.820 | 248.303 |
| Overdominant | CT *vs.* CC+TT | 1.624 (0.893-2.951) | 0.112 | 245.811 |
| Additive | T | 1.221 (0.796-1.874) | 0.361 | 247.520 |

OR: odds ratio; CI: confidence interval; AIC: Akaike information criteria; p-value: significance level (statistically significant when p < 0.05).

**Table S4. Distributions of *PTTG1* (rs1895320, rs2910200, rs3811999) genotypes and alleles in male patients with PitNET and the control group**

| **Gene** | **Genotype/Allele** | **PitNET group males (n=)**  **n (%)** | **Control group**  **males (n=)**  **n (%)** | ***p*-value** |
| --- | --- | --- | --- | --- |
| *PTTG1*  (rs1895320) | AA | 40 (80) | 81 (77.9) | 0.612 |
|  | AG | 10 (20) | 21 (20.2) |  |
|  | GG | 0 (0) | 2 (1.9) |  |
|  | In total: | 50 (100) | 104 (100) |  |
|  | Allele:  A  G | 90 (90)  10 (10) | 183 (88)  25 (12) | 0.601 |
| *PTTG1*  (rs2910200) | CC | 23 (46) | 51 (49) | 0.513 |
|  | CT | 19 (38) | 43 (41.3) |  |
|  | TT | 8 (16) | 10 (9.6) |  |
|  | In total: | 50 (100) | 104 (100) |  |
|  | Allele:  C  T | 65 (65)  35 (35) | 145 (69.7)  63 (30.3) | 0.405 |
| *PTTG1*  (rs3811999) | CC | 20 (40) | 38 (36.5) | 0.138 |
|  | CT | 19 (38) | 54 (51.9) |  |
|  | TT | 11 (22) | 12 (11.5) |  |
|  | In total: | 50 (100) | 104 (100) |  |
|  | Allele:  C  T | 59 (59)  41 (41) | 130 (62.5)  78 (37.5) | 0.554 |

**Table S5. Binary logistic regression analysis of *PTTG1* (rs1895320, rs2910200, rs3811999) in male patients with PitNET and control groups**

| **Model** | **Genotype/Allele** | **OR (95% CI)** | ***p*-value** | **AIC** |
| --- | --- | --- | --- | --- |
| ***PTTG1* (**rs1895320**)** | | | | |
| Codominant | AG *vs.* AA  GG *vs.* AA | 0.964 (0.415-2.240)  - | 0.933  - | 196.556 |
| Dominant | AG+GG *vs.* AA | 0.880 (0.383-2.026) | 0.765 | 196.055 |
| Recessive | GG *vs.* AA+AG | - | - | 194.563 |
| Overdominant | AG *vs.* AA+GG | 0.988 (0.426-2.294) | 0.978 | 196.145 |
| Additive | G | 0.813 (0.374-1.768) | 0.602 | 195.867 |
| ***PTTG1* (**rs2910200**)** | | | | |
| Codominant | CT *vs.* CC  TT *vs.* CC | 0.980 (0.472-2.034)  1.774 (0.620-5.079) | 0.956  0.286 | 196.866 |
| Dominant | CT+TT *vs.* CC | 1.130 (0.575-2.221) | 0.724 | 196.021 |
| Recessive | TT *vs.* CC+CT | 1.790 (0.660-4.859) | 0.253 | 194.869 |
| Overdominant | CT *vs.* CC+TT | 0.869 (0.435-1.736) | 0.692 | 195.988 |
| Additive | T | 1.222 (0.749-1.995) | 0.423 | 195.506 |
| ***PTTG1*** (rs3811999) | | | | |
| Codominant | CT *vs.* CC  TT *vs.* CC | 0.669 (0.315-1.419)  1.742 (0.653-4.646) | 0.294  0.268 | 194.275 |
| Dominant | CT+TT *vs.* CC | 0.864 (0.432-1.726) | 0.678 | 195.974 |
| Recessive | TT *vs.* CC+CT | 2.162 (0.879-5.317) | 0.093 | 193.376 |
| Overdominant | CT *vs.* CC+TT | 0.568 (0.285-1.130) | 0.107 | 193.499 |
| Additive | T | 1.158 (0.711-1.887) | 0.555 | 195.798 |

OR: odds ratio; CI: confidence interval; AIC: Akaike information criteria; p-value: significance level (statistically significant when p < 0.05).

**Table S6. Binary logistic regression analysis of *PTTG1* (rs1895320, rs2910200, rs3811999) in the PitNET and control groups by PitNET invasiveness**

| **Model** | **Genotype/Allele** | **OR (95% CI)** | ***p*-value** | **AIC** |
| --- | --- | --- | --- | --- |
| ***PTTG1* rs1895320** | | | | |
| **Non-invasive PitNET** | | | | |
| Codominant | AG *vs.* AA  GG *vs.* AA | 1.213 (0.611-2.407)  1.040 (0.209-5.171) | 0.581  0.962 | 303.793 |
| Dominant | AG+GG *vs.* AA | 1.188 (0.620-2.276) | 0.603 | 301.826 |
| Recessive | GG *vs.* AA+AG | 0.998 (0.202-4.927) | 0.998 | 302.092 |
| Overdominant | AG *vs.* AA+GG | 1.211 (0.612-2.396) | 0.583 | 301.796 |
| Additive | G | 1.125 (0.657-1.926) | 0.667 | 301.910 |
| **Invasive PitNET** | | | | |
| Codominant | AG *vs.* AA  GG *vs.* AA | 0.885 (0.413-1.898)  0.531 (0.064-4.426) | 0.754  0.559 | 285.133 |
| Dominant | AG+GG *vs.* AA | 0.835 (0.402-1.733) | 0.627 | 283.361 |
| Recessive | GG *vs.* AA+AG | 0.543 (0.065-4.508) | 0.572 | 283.233 |
| Overdominant | AG *vs.* AA+GG | 0.902 (0.421-1.930) | 0.790 | 283.530 |
| Additive | G | 0.826 (0.442-1.543) | 0.549 | 283.228 |
| ***PTTG1* rs2910200** | | | | |
| **Non-invasive PitNET** | | | | |
| Codominant | CT *vs.* CC  TT *vs.* CC | 1.536 (0.845-2.790)  1.327 (0.513-3.435) | 0.159  0.560 | 302.073 |
| Dominant | CT+TT *vs.* CC | 1.493 (0.844-2.639) | 0.168 | 300.167 |
| Recessive | TT *vs.* CC+CT | 1.071 (0.437-2.624) | 0.881 | 302.070 |
| Overdominant | CT *vs.* CC+TT | 1.453 (0.828-2.551) | 0.193 | 300.402 |
| Additive | T | 1.261 (0.835-1.905) | 0.271 | 300.890 |
| **Invasive PitNET** | | | | |
| Codominant | CT *vs.* CC  TT *vs.* CC | 1.017 (0.548-1.889)  1.016 (0.377-2.732) | 0.956  0.976 | 285.598 |
| Dominant | CT+TT *vs.* CC | 1.017 (0.568-1.821) | 0.955 | 283.598 |
| Recessive | TT *vs.* CC+CT | 1.008 (0.390-2.605) | 0.987 | 283.601 |
| Overdominant | CT *vs.* CC+TT | 1.015 (0.560-1.838) | 0.962 | 283.599 |
| Additive | T | 1.011 (0.654-1.562) | 0.960 | 283.599 |
| ***PTTG1* rs3811999** | | | | |
| **Non-invasive PitNET** | | | | |
| Codominant | CT *vs.* CC  TT *vs.* CC | 0.910 (0.497-1.666)  1.004 (0.423-2.382) | 0.759  0.993 | 303.981 |
| Dominant | CT+TT *vs.* CC | 0.931 (0.527-1.646) | 0.805 | 302.031 |
| Recessive | TT *vs.* CC+CT | 1.056 (0.472-2.358) | 0.895 | 302.075 |
| Overdominant | CT *vs.* CC+TT | 0.909 (0.517-1.596) | 0.739 | 301.981 |
| Additive | T | 0.977 (0.649-1.472) | 0.913 | 302.080 |
| **Invasive PitNET** | | | | |
| Codominant | CT *vs.* CC  TT *vs.* CC | 1.531 (0.780-3.003)  2.175 (0.924-5.118 | 0.215  0.075 | 282.220 |
| Dominant | CT+TT *vs.* CC | 1.676 (0.886-3.172) | 0.112 | 280.969 |
| Recessive | TT *vs.* CC+CT | 1.689 (0.802-3.555) | 0.167 | 281.791 |
| Overdominant | CT *vs.* CC+TT | 1.176 (0.657-2.107) | 0.585 | 283.303 |
| Additive | T | 1.481 (0.973-2.255) | 0.067 | 280.236 |

OR: odds ratio; CI: confidence interval; AIC: Akaike information criteria; p-value: significance level (statistically significant when p < 0.05).

**Table S7. Binary logistic regression analysis of *PTTG1* (rs1895320, rs2910200, rs3811999) in the PitNET and control groups by PitNET activity**

| **Model** | **Genotype/Allele** | **OR (95% CI)** | ***p*-value** | **AIC** |
| --- | --- | --- | --- | --- |
| ***PTTG1* rs1895320** | | | | |
| **Non-active PitNET** | | | | |
| Codominant | AG *vs.* AA  GG *vs.* AA | 0.947 (0.440-2.038)  - | 0.889  - | 269.639 |
| Dominant | AG+GG *vs.* AA | 0.812 (0.380-1.732) | 0.589 | 270.425 |
| Recessive | GG *vs.* AA+AG | - | - | 267.659 |
| Overdominant | AG *vs.* AA+GG | 0.986 (0.458-2.120) | 0.970 | 270.723 |
| Additive | G | 0.735 (0.372-1.453) | 0.735 | 269.887 |
| **Active PitNET** | | | | |
| Codominant | AG *vs.* AA  GG *vs.* AA | 1.140 (0.576-2.255)  1.466 (0.366-5.877) | 0.707  0.589 | 315.535 |
| Dominant | AG+GG *vs.* AA | 1.187 (0.629-2.240) | 0.598 | 313.643 |
| Recessive | GG *vs.* AA+AG | 1.426 (0.358-5.676) | 0.614 | 313.675 |
| Overdominant | AG *vs.* AA+GG | 1.119 (0.568-2.206) | 0.744 | 313.813 |
| Additive | G | 1.174 (0.703-1.961) | 0.541 | 313.552 |
| ***PTTG1* rs2910200** | | | | |
| **Non-active PitNET** | | | | |
| Codominant | CT *vs.* CC  TT *vs.* CC | 1.239 (0.665-2.306)  0.569 (0.158-2.044) | 0.500  0.387 | 270.995 |
| Dominant | CT+TT *vs.* CC | 1.100 (0.603-2.005) | 0.756 | 270.628 |
| Recessive | TT *vs.* CC+CT | 0.514 (0.148-1.780) | 0.294 | 269.450 |
| Overdominant | CT *vs.* CC+TT | 1.339 (0.733-2.448) | 0.342 | 269.827 |
| Additive | T | 0.945 (0.596-1.498) | 0.809 | 270.666 |
| **Active PitNET** | | | | |
| Codominant | CT *vs.* CC  TT *vs.* CC | 1.283 (0.711-2.315)  1.693 (0.723-3.963) | 0.408  0.225 | 314.277 |
| Dominant | CT+TT *vs.* CC | 1.368 (0.787-2.377) | 0.267 | 312.675 |
| Recessive | TT *vs.* CC+CT | 1.503 (0.676-3.340) | 0.318 | 312.961 |
| Overdominant | CT *vs.* CC+TT | 1.145 (0.658-1.991) | 0.632 | 313.689 |
| Additive | T | 1.296 (0.873-1.924) | 0.198 | 312.279 |
| ***PTTG1* rs3811999** | | | | |
| **Non-active PitNET** | | | | |
| Codominant | CT *vs.* CC  TT *vs.* CC | 1.689 (0.837-3.409)  2.279 (0.934-5.560) | 0.143  0.070 | 268.924 |
| Dominant | CT+TT *vs.* CC | 1.822 (0.935-3.553) | 0.078 | 267.440 |
| Recessive | TT *vs.* CC+CT | 1.659 (0.770-3.573) | 0.196 | 269.140 |
| Overdominant | CT *vs.* CC+TT | 1.272 (0.698-2.320) | 0.432 | 270.106 |
| Additive | T | 1.527 (0.989-2.358) | 0.056 | 267.055 |
| **Active PitNET** | | | | |
| Codominant | CT *vs.* CC  TT *vs.* CC | 0.875 (0.484-1.582)  1.036 (0.450-2.382) | 0.658  0.934 | 315.652 |
| Dominant | CT+TT *vs.* CC | 0.911 (0.523-1.588) | 0.743 | 313.810 |
| Recessive | TT *vs.* CC+CT | 1.111 (0.512-2.410) | 0.790 | 313.848 |
| Overdominant | CT *vs.* CC+TT | 0.867 (0.500-1.504) | 0.611 | 313.659 |
| Additive | T | 0.980 (0.658-1.460) | 0.920 | 313.908 |

OR: odds ratio; CI: confidence interval; AIC: Akaike information criteria; p-value: significance level (statistically significant when p < 0.05).

**Table S8. Binary logistic regression analysis of *PTTG1* (rs1895320, rs2910200, rs3811999) in the PitNET and control groups by PitNET recurrence**

| **Model** | **Genotype/Allele** | **OR (95% CI)** | ***p*-value** | **AIC** |
| --- | --- | --- | --- | --- |
| ***PTTG1* rs1895320** | | | | |
| **PitNET without recurrence** | | | | |
| Codominant | AG *vs.* AA  GG *vs.* AA | 1.115 (0.613-2.030)  1.004 (0.253-3.990) | 0.721  0.996 | 391.950 |
| Dominant | AG+GG *vs.* AA | 1.100 (0.624-1.937) | 0.743 | 389.971 |
| Recessive | GG *vs.* AA+AG | 0.982 (0.248-3.879) | 0.979 | 390.077 |
| Overdominant | AG *vs.* AA+GG | 1.115 (0.614-2.025) | 0.720 | 389.951 |
| Additive | G | 1.065 (0.666-1.703) | 0.792 | 390.009 |
| **PitNET with recurrence** | | | | |
| Codominant | AG *vs.* AA  GG *vs.* AA | 0.814 (0.264-2.509)  - | 0.720  - | 159.271 |
| Dominant | AG+GG *vs.* AA | 0.698 (0.228-2.138) | 0.529 | 158.452 |
| Recessive | GG *vs.* AA+AG | - | - | 155.403 |
| Overdominant | AG *vs.* AA+GG | 0.848 (0.275-2.611) | 0.773 | 158.790 |
| Additive | G | 0.653 (0.237-1.803) | 0.411 | 158.112 |
| ***PTTG1* rs2910200** | | | | |
| **PitNET without recurrence** | | | | |
| Codominant | CT *vs.* CC  TT *vs.* CC | 1.129 (0.679-1.875)  1.053 (0.464-2.390) | 0.641  0.901 | 391.860 |
| Dominant | CT+TT *vs.* CC | 1.113 (0.689-1.799) | 0.662 | 389.887 |
| Recessive | TT *vs.* CC+CT | 0.996 (0.455-2.182) | 0.992 | 390.078 |
| Overdominant | CT *vs.* CC+TT | 1.118 (0.688-1.818) | 0.652 | 389.876 |
| Additive | T | 1.061 (0.742-1.517) | 0.747 | 389.974 |
| **PitNET with recurrence** | | | | |
| Codominant | CT *vs.* CC  TT *vs.* CC | 2.013 (0.799-5.073)  1.777 (0.438-7.214) | 0.138  0.421 | 158.515 |
| Dominant | CT+TT *vs.* CC | 1.964 (0.807-4.777) | 0.137 | 156.549 |
| Recessive | TT *vs.* CC+CT | 1.224 (0.339-4.421) | 0.758 | 158.784 |
| Overdominant | CT *vs.* CC+TT | 1.773 (0.760-4.135) | 0.185 | 157.115 |
| Additive | T | 1.475 (0.806-2.697) | 0.207 | 157.318 |
| ***PTTG1* rs3811999** | | | | |
| **PitNET without recurrence** | | | | |
| Codominant | CT *vs.* CC  TT *vs.* CC | 1.009 (0.596-1.709)  1.369 (0.673-2.787) | 0.974  0.386 | 391.225 |
| Dominant | CT+TT *vs.* CC | 1.090 (0.665-1.786) | 0.732 | 389.960 |
| Recessive | TT *vs.* CC+CT | 1.363 (0.711-2.611) | 0.351 | 389.226 |
| Overdominant | CT *vs.* CC+TT | 0.922 (0.569-1.492) | 0.740 | 389.968 |
| Additive | T | 1.135 (0.804-1.602) | 0.471 | 389.559 |
| **PitNET with recurrence** | | | | |
| Codominant | CT *vs.* CC  TT *vs.* CC | 1.971 (0.726-5.347)  1.933 (0.511-7.321) | 0.183  0.332 | 158.826 |
| Dominant | CT+TT *vs.* CC | 1.962 (0.749-5.139) | 0.170 | 156.827 |
| Recessive | TT *vs.* CC+CT | 1.267 (0.405-3.962) | 0.685 | 158.717 |
| Overdominant | CT *vs.* CC+TT | 1.590 (0.677-3.734) | 0.287 | 157.724 |
| Additive | T | 1.450 (0.789-2.665) | 0.232 | 157.454 |

OR: odds ratio; CI: confidence interval; AIC: Akaike information criteria; p-value: significance level (statistically significant when p < 0.05).
